# Force Fields and Simulation Engines: Lessons Learned from Porting Amber ff14SB and ff19SB to GROMACS

**DOI:** 10.1021/acs.jctc.6c01592

**Published:** 2026-10-20

**Authors:** Vedran Miletić, Miłosz Wieczór, Carlos Simmerling, Markus Rampp, Bert L. de Groot, Carsten Kutzner, Vytautas Gapsys

**Affiliations:** † 531612 Max Planck Computing and Data Facility, Gießenbachstraße 2, 85748 Garching, Germany; ‡ Institute for Research in Biomedicine (IRB Barcelona), The Barcelona Institute of Science and Technology, Baldiri Reixac 10−12, 08028 Barcelona, Spain; § Department of Physical Chemistry, Gdańsk University of Technology, Narutowicza 11/12, 80-233 Gdańsk, Poland; ∥ Department of Chemistry and Laufer Center for Physical and Quantitative Biology, 12301 Stony Brook University, Stony Brook, New York 11794, United States; ⊥ Department of Theoretical and Computational Biophysics, 28282 Max Planck Institute for Multidisciplinary Sciences, Am Faßberg 11, 37077 Göttingen, Germany; # 50148 Johnson and Johnson Innovative Medicine, Turnhoutseweg 30, 2340 Beerse, Belgium

## Abstract

The GROMACS molecular dynamics simulation package has historically supported a number of molecular mechanics force fields, including Amber. Force fields have evolved over time and continue to do so through refinement of existing interactions and the definition of additional ones to more faithfully match real-world experiments and higher-level quantum chemistry calculations. Although users have provided a number of conversions of newer Amber force fields over the years, the two most recent Amber protein force fields, ff14SB and ff19SB, had not previously been officially ported to GROMACS. A technical challenge is that ff19SB introduces residue-specific backbone dihedral energy correction maps (CMAPs), which required new support in GROMACS for residue-aware force-field preprocessing and topology generation. Unlike older force fields such as CHARMM27, in which CMAPs are parameterized using only atom types, ff19SB extends this scheme with additional amino-acid typing. Here, we describe how we ported and validated the Amber ff14SB and ff19SB force fields for use with GROMACS as AMBER14SB and AMBER19SB, respectively. The ported force fields reproduce the Amber reference topologies exactly, with per-interaction energies matching to within the single-precision floating-point limit (<10^–7^ kJ/mol). To achieve this level of consistency between Amber and GROMACS, we resolved discrepancies in their handling of fundamental constants, parameter precision, and improper-dihedral atom ordering logic. In long unrestrained ubiquitin simulations, the converted ff19SB reproduces the conformational free-energy profile obtained with the native Amber implementation. This work provides the first official port of an Amber force field to GROMACS in more than a decade, improving feature parity between the two simulation packages. We provide a reproducible porting and validation procedure that can be used for modified variants and future versions of Amber force fields.

## Graphical abstract

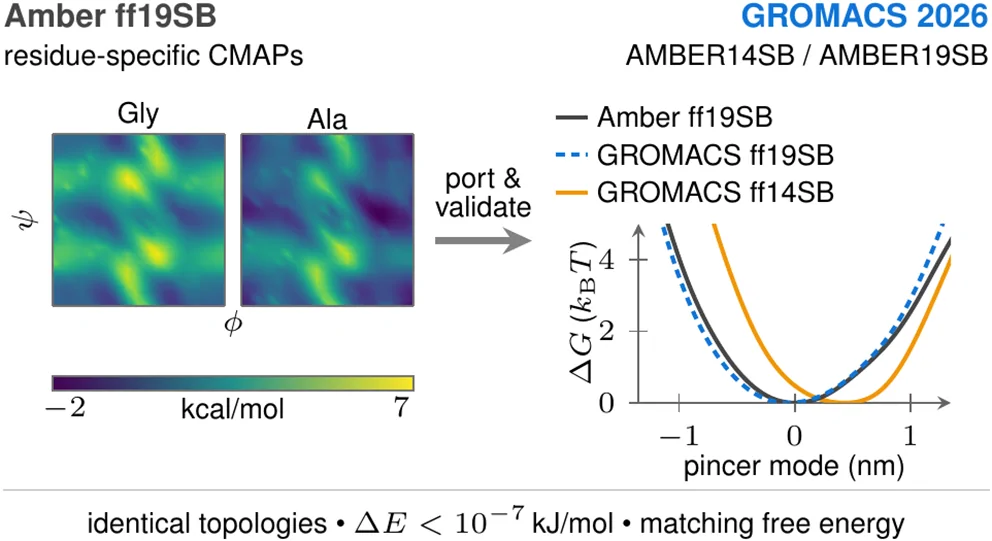

## 1 Introduction

At the core of every molecular dynamics (MD) simulation is a force field (FF) that defines the potential energy for interactions between particles. CHARMM^1,2^ and Amber^3–5^ are popular FF families for biomolecular simulations. They originate from academic software packages with the same name, and are subject to updates and refinements over time. These force fields have been ported for use with other MD packages, such as GROMACS,^6^ OpenMM,^7^ and LAMMPS.^8^

Until recently, GROMACS lagged behind in providing validated, officially supported conversions of recent Amber and CHARMM FFs. CHARMM addresses this issue by offering the latest version of the CHARMM36 FF in a GROMACS-ready file format.^9^ However, the same is not true for Amber. Amber permits the porting of its FFs to other packages, but it does not officially endorse these third-party ports (e.g., ports of its recent force fields for OpenMM are created, maintained, and supported by OpenMM developers^7^). The most recent Amber FF whose conversion has been validated and is officially supported by GROMACS is ff03; chronologically later derivatives of ff99, such as ff99SB^10^ from 2006 and ff99SB-ILDN^11^ from 2010, are also shipped with GROMACS, but without the same level of validation. Until now, newer FFs, including ff14SB^4^ and ff19SB^5^ were not included in GROMACS. To address this issue, in the current work, we present our ports of the two most recent Amber FFs, ff19SB and ff14SB.

An alternative solution to the native FF ports in GROMACS, could be file-format conversion directly from a topology generated in Amber for every new moiety.^12–14^ This route, however, would require preparing Amber topology first, and, subsequently using a converter. The goal of this work is instead to provide a native GROMACS implementation that generates topologies directly from the Amber force-field parameters, without relying on additional conversion tools. This avoids extra dependencies and allows us to validate the port directly at the parameter and energy level. We also note that current converters can be unreliable for modern force fields such as ff19SB, in some cases silently losing residue-specific CMAP terms.

Porting Amber ff19SB to GROMACS had one major prerequisite: support for grid-based energy correction maps (CMAPs) that are amino-acid-specific. The CMAP energy term was introduced in CHARMM22/CMAP^1,15^ to correct the two-dimensional energy surface of the ϕ, ψ protein backbone dihedral angles to better match quantum mechanical (QM) potential energy maps and experimental data.

In the ff19SB protein FF, the CMAPs were further refined. Unlike earlier CHARMM FFs, which relied on several generalized CMAPs, ff19SB derives CMAP specifically for each amino acid from high-level QM torsion scans performed on acetyl- and *N*-methyl-capped dipeptides (Ace-X-Nme), allowing the model to capture subtle sequence-dependent variations in backbone preferences. This residue-specific treatment improves the representation of secondary-structure propensities and enhances agreement with experimental observables.

Because integrating a new, state-of-the-art FF into GROMACS is valuable to many users, we aimed to make the conversion and validation process as automated and reproducible as possible. This approach should enable the reliable conversion of modified Amber FFs and future official Amber FF releases. The validation procedure ensures that the parameter conversion is correct and that single-point energies across a peptide library match between Amber and GROMACS. In addition, we compared conformational ensembles of ubiquitin generated in long-timescale MD simulations in both simulation engines. In such a comparison, obtaining identical ensembles is complicated by numerous differences in the algorithmic details of the two MD packages, e.g., neighbor-list construction, dynamic cutoff adjustment, bond constraints, and pressure-coupling methods. The ensembles generated with the Amber and GROMACS engines, however, are expected to match within the range of statistical uncertainty.

In the current work, we present an automated workflow for porting protein force fields from Amber to GROMACS. We have converted the two most recent Amber FFs, ff14SB and ff19SB. The ported FFs are included in the official GROMACS 2026 release, and the amino-acid-specific CMAP support on which ff19SB depends has been available since GROMACS 2025; the corresponding release information is summarized in Section 2.4. We systematically validated the FF ports and identified several discrepancies between the Amber and GROMACS simulation engines; we resolve and describe these differences in detail in this work.

## 2 Methods

### 2.1 Per-Amino-Acid CMAPs

Prior to version 2025, GROMACS support for CMAPs was limited to the CHARMM FF, where CMAP types are assigned by atom-type matching. For ff19SB, this support had to be extended to consider residue types in addition to atom types when assigning CMAP types. To allow flexible CMAP assignment, the original format:

[cmaptypes]

C N XC C N 1 24 24

–1.6941016 −3.83099592 −2.76190024 −0.5186068 (...)

was extended to include the residue type with each atom type:

[ cmaptypes ]

C-* N-ALA XC-ALA C-ALA N-* 1 24 24

–1.6941016 −3.83099592 −2.76190024 −0.5186068 (...)

This allows CMAP types to be specified for side chains or ligands, if such needs arise in the future.

### 2.2 Dihedral Atom Reordering

Amber’s topology preprocessor LEaP reorders atoms in improper dihedrals alphabetically by atom type. Initially introduced to speed up parameter lookup, this feature was kept afterward for compatibility and, eventually, for historical reasons. In the following, we assume that four atoms *a*_*i*_, *a*_*j*_, *a*_*k*_, *a*_*l*_ have types *t*_*i*_, *t*_*j*_, *t*_*k*_, *t*_*l*_, respectively. The reordering is performed lexicographically based on the atom types and is done in two steps.

In the first step, an improper dihedral defined in the topology by the atoms (*a*_*i*_, *a*_*j*_, *a*_*k*_, *a*_*l*_) may be reordered to (*a*_*l*_, *a*_*k*_, *a*_*j*_, *a*_*i*_) if *t*_*i*_ > *t*_*l*_ or (*t*_*i*_ = *t*_*l*_ and *t*_*j*_ > *t*_*k*_), where the greater-than operator is considered in the context of lexicographical ordering. The atom types are taken from the matching dihedral type, except in the case of the wildcard type X, which can correspond to any atom type and is replaced by a space for the reordering logic. Therefore, the wildcard type comes first in lexicographical ordering.

In the second step, the atoms are shuffled further. The third atom (*a*_*k*_ or *a*_*j*_, depending on the ordering from the first step) is taken to be central. The remaining atoms (*a*_*i*_, *a*_*j*_, *a*_*l*_ or *a*_*l*_, *a*_*k*_, *a*_*i*_, depending on the ordering from the first step) are then ordered lexicographically by atom type from the matching improper dihedral type. The second step of reordering is not applied to the first occurrence of that improper dihedral type in the topology, i.e., only occurrences from the second onward get their atoms reordered.

As an example, consider four atoms (a_20_, *a*_24_, *a*_22_, *a*_23_) with types C, XC, N, and H, forming an improper dihedral of the matching type C XC N H. This ordering would be used for calculating the improper dihedral energy at the first occurrence in the topology, but starting from the second occurrence, the following shuffling is performed: the central atom *a*_22_ (type N) is fixed in the third position, while the remaining atoms *a*_20_, *a*_24_, and *a*_23_ are ordered lexicographically by type, that is, as C, H, XC. The resulting improper dihedral is therefore (a_20_, *a*_23_, *a*_22_, *a*_24_).

Changing the order of atoms in a dihedral results in different definitions of the planes between which an angle is calculated. Therefore, atom reordering in an improper dihedral affects interaction energies and forces. We have implemented functionality in the GROMACS topology preprocessing that enables the use of Amber’s original improper dihedral atom reordering. This feature is optional: it is switched on by default for the newly ported ff14SB and ff19SB FFs. Disabling this option makes the topology preprocessor follow the atom ordering defined in the topology file: this is the standard behavior in GROMACS.

### 2.3 Force Field Conversion

GROMACS and Amber packages follow different force-field organizational schemes. Therefore, porting a FF is not a one-to-one file-format conversion; instead, it requires extracting information from the Amber format and reorganizing it within the GROMACS FF infrastructure. Manual conversion is slow, error-prone, tedious, and difficult to reuse for future FF ports. Therefore, we explored options for automating the conversion.

As the Gromologist library^16^ provides many functions for reading and writing Amber and CHARMM FFs in GROMACS format, we decided to use it as a starting point for our workflow. Gromologist reads a specified leaprc file, deduces which other files are referenced and need to be read, transforms the parameters, and arranges them into files in a specified GROMACS FF directory. We have refined the Gromologist tool to make the conversion process more reproducible, resolved some minor formatting issues, and increased the number of digits after the decimal point in the output files. Our modified version is available at https://gitlab.mpcdf.mpg.de/vedm/gromologist.

The conversion procedure populates the specified directory with the files containing the converted atom types, parameters for bonded and nonbonded interaction types, and amino-acid residue parameters. The other files required for the FF are copied from an existing GROMACS FF (e.g., Amber ff99), if available on the path. If the required files are not available, the copying is skipped and the converted FF is not expected to be usable in GROMACS as-is; this mode of operation is intended for conversion functionality development and library debugging.

Due to inherent limitations of the automated conversion, the files in the populated GROMACS FF directory required the following manual changes:

- addition or adjustment of residue and atom names in the files copied from the existing FF (notably, atom names were adjusted to match those in the current Amber FF),
- addition of a missing atom type (CJ in case of ff14SB and ff19SB),
- addition of separately converted water models together with corresponding ions for each water model,
- addition of missing improper dihedrals to amino-acid residues and their associated improper types to the list of dihedral types,
- reordering of incorrectly ordered improper dihedrals in residues and their associated improper types in the list of types.

The OPC and OPC3 water models were converted for inclusion in GROMACS. Other water models supported by ff14SB and ff19SB that were previously available in GROMACS format for other Amber FFs (TIP3P, TIP4Pew, SPC, and SPC/E) were converted anew.

### 2.4 Software Versions

The functionality described above was introduced incrementally in different GROMACS releases. Amino-acid-specific CMAP assignment, i.e., the ability to assign CMAP types using residue types in addition to atom types, was introduced in GROMACS 2025 and is present in all subsequent releases.^17^ Most of the remaining developments reported here, together with the ported FFs, first appear in GROMACS 2026, either in the initial release or, in one case, in a later 2026.x patch release (Table 1).^18^ The one exception is the Amber-compatible electric conversion factor discussed in the Results: this patch has been proposed but is not yet part of an official GROMACS release.

**Table 1:** First GROMACS Release for Each Feature

feature	release
Amino-acid-specific CMAP types (keyed by atom and residue types)	2025.0
Amber LEaP-compatible improper-dihedral atom reordering	2026.0
Automatic residue renaming for CMAPs (pdb2gmx -rtpres auto)	2026.0
Water-model-aware ion selection (gmx genion)	2026.0
Bundled amber14sb and amber19sb FFs (including OPC and OPC3)	2026.0
Higher-precision 1–4 electrostatic scaling (fudgeQQ)	2026.2
Amber-compatible electric conversion factor ([defaults])	proposed

In practice this means that GROMACS 2026 or later is required to use the ported ff14SB and ff19SB FFs as distributed, because the FF directories and the LEaP-compatible dihedral reordering they rely on are part of that release. GROMACS 2025 or later can read the amino-acid-specific CMAP format, which is relevant for users who construct such a FF themselves, but it does not ship the FF ports.

### 2.5 Validation

We validated the FF port in two stages: (1) comparing peptide topologies and (2) comparing peptide energies produced by the Amber and GROMACS packages. During the first stage, the prepared inputs for the MD simulations were compared in terms of interaction parameters that constitute the molecular topology. During the second stage, the MD simulation output was compared in terms of bonded and nonbonded interaction energies of the molecule, decomposed by interaction type. The validation workflow was fully automated.

We offer a detailed description of this workflow, depicted in Figure 1, in the following subsections.

**Figure 1:**
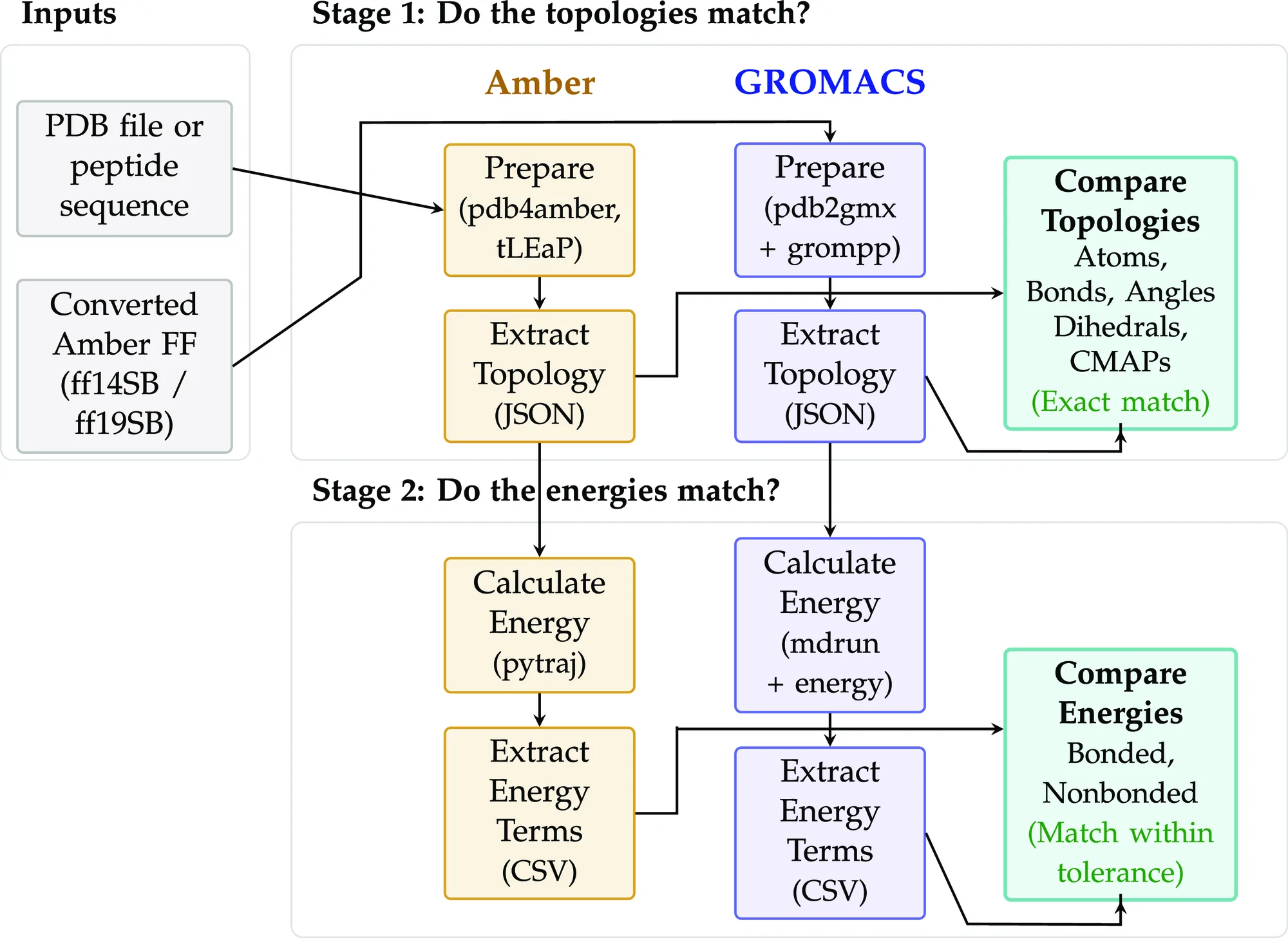
To validate a converted FF, the same molecular input is processed with the Amber and GROMACS engines, and both molecular topologies and computed energies are extracted and compared. Validation succeeds when topologies are identical and energies match within numerical precision.

### 2.6 Common Parts of the Topology and Energy Comparison Workflows

The workflow starts from either a structure file in PDB format or a peptide sequence in textual format and finishes with the results of the comparisons stored in text files. Intermediate output and input files are kept in the scratch directory to enable step-by-step posthoc debugging of the workflow. Additionally, the standard output and standard error streams from all commands and scripts executed by the workflow are kept in the logs directory after execution.

If the input is a file in PDB format, it can be optionally accompanied by a trajectory in XTC or NetCDF format, and a specified number of frames will be extracted from the trajectory. All the extracted frames will then be processed independently as individual PDB files. The Amber program pdb4amb er is used to convert the PDB into an Amber-compatible structure file.

If the input is a peptide sequence in text format, it can be specified in two distinct ways:

- one-letter amino-acid symbols, without spaces between them,
- three-letter amino-acid symbols, with dashes between them.

In the latter case, explicitly specifying the LEaP residue types in addition to the standard three-letter symbols is also possible. In both cases, the result is saved into a PDB file and energy minimization is performed to relax the initial conformation.

The rest of the input preparation includes the following tasks:

- first pass of LEaP to get a PDB file with Amber-compatible naming,
- gmx editconf command to place the molecule in the center of a cubic box of the specified size,
- second pass of LEaP to obtain Amber input coordinates and parameter files,
- gmx pdb2gmx command to obtain GROMACS input coordinates and parameter files,
- optionally, if a bond, angle, dihedral, or CMAP interaction is specified, all other interactions of the same type are removed from the Amber and GROMACS topologies using ParmEd; this way, if desired, the comparison of molecular topologies or energies can focus on a single interaction at a time,
- execution of custom scripts that rename atoms to match either Amber or GROMACS naming conventions and remove solvent molecules (using ParmEd).

### 2.7 Topology Comparison

Prepared topologies are read by ParmEd and saved in a specified format (either Amber or GROMACS). Additionally, for the GROMACS topology, the LEaP reordering feature is used in the gmx grompp command to ensure that the order of atoms in (proper and improper) dihedrals matches that of Amber. Since this command does not output the final topology files obtained after LEaP reordering, its effects are reconstructed from the verbose command output. Afterward, the original topology file is modified accordingly using ParmEd. Atoms and bonded interactions are also exported in a custom JSON format.

The final results of the topology comparison produced by the workflow are two files in JSON format, specifically listing:

- individual bonded interactions, grouped by type, that are present in the Amber topology but absent in the GROMACS topology,
- individual bonded interactions, grouped by type, that are present in the GROMACS topology but absent in the Amber topology.

The results of the topology comparison were used for the iterative refinement of the converted FF.

### 2.8 Energy Comparison

We computed single-point energies for the energy-minimized peptides using Amber and GROMACS. For energy calculations and decomposition by interaction type in Amber, we used the pytraj software package^19^ with default settings, except that the generalized Born solvation model was disabled (igb = 0).^20,21^

For GROMACS calculations, we used a plain cutoff of 10 nm for electrostatic and van der Waals interactions, large enough to encompass all interactions within the box. A cubic box with an edge length of 25 nm was used to avoid spurious interactions through periodic boundaries. No constraints on bonds were applied. The calculations were performed in double precision.

In the comparison phase, pytraj energy terms were first mapped to the corresponding terms in GROMACS, and then the absolute difference in their values was calculated. Optionally, the average per-interaction energy differences for each term were calculated by normalizing the absolute difference by the corresponding interaction count, which was extracted from the GROMACS topology. The choice of GROMACS topology assumed that the number of interactions in GROMACS and Amber topologies was the same. Otherwise, i.e., if a topology mismatch between the engines was present, specifically in the number of interactions contributing to a given energy term, then an energy mismatch was registered for the corresponding term. Consequently, matching the topologies between the MD engines for a particular molecule was a prerequisite for matching the energies.

### 2.9 Implementation Details

To automate the sequence of operations in the order described above, we used Snakemake, a scalable workflow engine.^22^ Snakemake workflow definitions are written in a human-readable format that specifies the external commands or scripts for execution together with their input and output files. Snakemake handles task input, output, and resource requirements and uses this information to order the tasks for execution, possibly in parallel, if no interdependencies exist. Workflow configuration is read from a YAML file, with optional overrides using command line parameters. Notably, Snakemake workflows are redistributable and reproducible, which aligns with the goals of this effort.

Aside from Snakemake, GROMACS, and AmberTools, the workflow optionally can make use of PyMOL^23^ for building peptides (using LEaP for the same purpose is also possible), MDTraj^24^ for extracting frames from GROMACS trajectories, SciPy’s^25^ NetCDF reader for extracting frames from Amber trajectories, and Matplotlib^26^ for plotting.

In addition, we made the following modifications to ParmEd:

- support for per-amino-acid CMAPs. This change was necessary because unmodified ParmEd would collapse the residue-specific CMAP terms of ff19SB onto a single generic type,
- two new entries in the defaults section of GROMACS topologies,
- increased the output file precision.

Our modified variant of ParmEd is available at https://gitlab.mpcdf.mpg.de/vedm/parmed. The source code of the force field validation workflow is available under the terms of the AGPLv3 license at https://gitlab.mpcdf.mpg.de/barnsteen/workflow.

### 2.10 Validation Sets

We constructed three validation sets to probe different aspects of the ported force fields. The set of tetrapeptides contains all possible placements of amino acid pairs in a sequence surrounded or interspaced by alanines. This set was used to compare point energies in a local potential energy minimum.

Another validation system, 27 amino acid peptide, contained all amino acid types in a single sequence. We used this system to compare energies from MD simulations at various temperatures.

A protein ubiquitin was selected as the third validation system. The free-energy landscape of this protein is well understood and characterized^27^ making it a convenient system for long-timescale MD simulations, from which the resulting free-energy profiles can be used to validate force field conversion.

#### 2.10.1 Tetrapeptides

The tetrapeptide set includes sequences of type AxyA and xAAy, where x and y are any of the 20 natural amino acids, and contains 799 molecules in total. Each amino acid appears in the set as N-terminal, C-terminal, and nonterminal. We chose to use tetrapeptides instead of shorter sequences for validation due to the specifics of atom reordering in improper dihedrals by the Amber package. The appearance of a particular amino acid in the third position (i.e., second nonterminal position) instead of the second (i.e., first nonterminal) might result in different improper dihedral types being assigned to it, because atom reordering is applied only to the second and later appearances of the same dihedral in the topology.

#### 2.10.2 27-Amino-Acid-Peptide Conformations

The 27-peptide contains all amino acids supported by ff14SB and ff19SB in the nonterminal configurations (its N-terminal and C-terminal amino acids are alanines): all natural amino acids and their alternative protonation forms. The set of the peptide’s conformations was obtained by running two independent 100 ns MD simulations (solvated in TIP3P water,^28^ neutralized by adding 0.15 M Na^+^ and Cl^–^ ions), one at 298 K and one at 498 K, and extracting 1000 frames from each. The purpose of the high-temperature ensemble is to observe the impact of sampling higher energetic barriers on the energy differences between MD engines.

#### 2.10.3 Ubiquitin

The ubiquitin (acetyl- and *N*-methylamide-capped) structure 1UBQ^29^ was prepared with ff19SB and solvated with TIP3P water molecules in a cubic box with an initial edge length of 7.58 nm. Sodium and chloride ions were added at a concentration of 0.15 M (39 Na^+^ and 39 Cl^–^). MD simulations were then performed independently with Amber and GROMACS. The two simulation engines were configured to match as closely as possible in terms of cutoffs, PME parameters, temperature and pressure control, and constraint schemes, to enable direct comparison of free-energy profiles.

Ubiquitin simulations were performed with Amber and GROMACS simulation engines by running 10 independent replicas of 10 μs each.

#### 2.10.4 GROMACS Simulation Parameters

Each of the ten replicas was propagated with the leapfrog integrator (integrator = md) for 5 × 10^9^ steps (10 μs) under three-dimensional periodic boundary conditions (pbc = xyz). PME^30^ with a real-space cutoff of 1 nm was used to treat electrostatic interactions. Spline interpolation was set to fourth order, and the real-space tolerance was set to 10^–5^. A potential-shift modifier was used for the van der Waals interactions; the cutoff was set to 1 nm. Long-range van der Waals interactions beyond the cutoff were accounted for with the analytic dispersion correction to energy and pressure (DispCorr = EnerPres). Temperature was maintained at 298 K using the velocity-rescaling thermostat^31^ (τ_*T*_ = 1.0 ps). Pressure was controlled isotropically at 1 bar using the C-rescale barostat^32^ (τ_*P*_ = 5.0 ps, compressibility 4.6 × 10^–5^ bar^–1^). All bonds involving hydrogen atoms were constrained using the LINCS algorithm^33^ (order four, two iterations), enabling a time step of 2 fs. Center-of-mass motion of the whole system was removed every 100 steps (comm-mode = Linear, nstcomm = 100). Compressed trajectory frames were saved every 10^5^ steps.

#### 2.10.5 Amber Simulation Parameters

The simulations with Amber were performed using the pmemd module.^34^ The full 5 × 10^9^-step (10 μs) production for ten replicas, matching the GROMACS run length, was split into consecutive runs of 5 × 10^8^ steps (1 μs) each, because Amber stores the number of steps of a single execution as a 32-bit integer and therefore cannot cover the whole trajectory in one run. For the first run, each replica was initiated from coordinates only (ntx = 1, irest = 0), with velocities assigned at the target temperature from an independently drawn random seed (ig = −1), making the replicas independent. Afterward, simulations were restarted using saved coordinates and velocities (ntx = 5, irest = 1). A real-space cutoff of 10 Å (1.0 nm) was applied for both Lennard-Jones and short-range electrostatic interactions. Long-range electrostatics were treated using PME with a fourth-order B-spline interpolation (order = 4) and a real-space Ewald tolerance of 10^–5^. Long-range van der Waals interactions beyond the cutoff were accounted for with the analytic dispersion correction to energy and pressure (vdwmeth = 1). Temperature was maintained at 298 K using the stochastic velocity-rescaling thermostat (ntt = 11) with a 1.0 ps temperature-coupling time (tautp = 1.0). Pressure was controlled isotropically at 1 bar under constant-pressure periodic boundary conditions (ntb = 2) using the Monte Carlo barostat^35^ (ntp = 1, barostat = 2) with a relaxation time of 5.0 ps and a compressibility of 4.6 × 10^–5^ bar^–1^. All bonds involving hydrogen atoms were constrained (ntc = 2, ntf = 2) with the SHAKE^36^ algorithm using a tolerance of 10^–4^, enabling a 2 fs time step (dt = 0.002). Center-of-mass motion of the whole system was removed every 100 steps (nscm = 100). Coordinates and velocities were written every 10^5^ steps.

## 3 Results and Discussion

The current work delivers two central results: a general workflow for converting modern Amber family FFs into a GROMACS-compatible format, and, as a specific result, the converted and validated ff14SB and ff19SB force fields. To verify the correctness of the conversion, we performed multiple consistency checks and systematically compared interaction energies computed by GROMACS with those from the Amber simulation engine. These analyses revealed subtle differences between both MD engines, which we subsequently resolved to enable reliable FF conversion. We then compared the conformational free-energy profiles of ubiquitin obtained with Amber using ff19SB to those obtained with GROMACS using the converted ff19SB.

### 3.1 Force Field Conversion and Amino-Acid-Specific CMAP Correction

We developed an automated toolkit for converting Amber protein FFs into a GROMACS-compatible format. It includes the automated conversion of atom types, their associated nonbonded (van der Waals, partial charges), and bonded (bonds, angles, dihedrals, CMAP) parameters. While this strategy is sufficient for porting earlier generations of Amber FFs, ff19SB presented an additional challenge: CMAP definitions are specific to each amino acid.

CMAP interactions have been available in GROMACS since 2010 to support the CHARMM family of FFs.^37^ However, the implementation was limited to defining a CMAP grid for each unique combination of five atoms. This approach was sufficient for CHARMM FFs, where all canonical amino acids except proline and glycine use the same correction map. Thus, the five-atom sequence C NH1 CT1 C NH1 maps to the same CMAP grid for these 18 amino acids, while glycine and proline have different names for their backbone atoms, allowing each to be assigned a different CMAP grid.

The situation is different for ff19SB, where each amino acid requires a unique CMAP correction. For example, assigning different correction grids to alanine and valine was not possible with the legacy GROMACS CMAP implementation: both amino acids have the same backbone atom definition C N XC C N, even though they must map to different CMAPs. One possible workaround is to give each amino acid a unique name for its backbone atoms; this approach is used by CHARMM-GUI.^38^ This, however, introduces unnecessary atom-type redundancies and is an error-prone practice.

We took a different route and introduced changes in GROMACS 2025 that enable flexible CMAP definitions. For the example above, the correction grid for alanine is now defined as C-* N-ALA XC-ALA C-ALA N-–*, while for valine it is C-* N-VAL XC-VAL C-VAL N-*, leaving no ambiguity in CMAP assignment. We also ensured backward compatibility with the former amino-acid-agnostic CMAP definition.

### 3.2 Water Models and Ions

The current FF port includes water-model and ion conversions that are consistent with Amber ff14SB and ff19SB as implemented in Amber. The converted water models include TIP3P,^28^ TIP4Pew,^39^ SPC,^40^ SPC/E,^41^ OPC,^42^ and OPC3.^43^ Although these water models were originally designed to be rigid, the FF defines bond and angle force constants. We therefore took these bond and angle force constants directly from the original Amber force-field parameter files (as distributed with Amber, e.g., the LEaP parm files).

Ion parameters were ported consistently with their representation in Amber; that is, each water model has a corresponding ion parameter set.^44^ This feature also required modifications in GROMACS: when used with the latest ff14SB and ff19SB FFs, the gmx genion tool recognizes the selected water model and chooses the appropriate ion model.

### 3.3 Matching Topologies between Amber and GROMACS

A prerequisite for matching computed energies between the MD engines is that the molecular topologies produced by Amber LEaP and GROMACS pdb2gmx from the same input agree for every interaction. After applying the conversion and the dihedral-reordering fix described in Section 2.2, the Amber and GROMACS topologies for the tetrapeptide and 27-peptide test sets match in the number, type, and atom membership of every bonded interaction (bonds, angles, dihedrals, and CMAPs), with the corresponding parameter values agreeing to at least six decimal digits. This level of agreement is a prerequisite for the per-interaction energy comparison in the following subsection. If there is any topology mismatch (e.g., a missing interaction or an incorrectly ordered improper dihedral), the corresponding energy term would also differ between the engines, and the comparison would not be a valid test of the converted FF.

### 3.4 Matching Interaction Energies between Amber and GROMACS

First, the ported ff19SB FF was evaluated on a set of tetrapeptides in the Amber and GROMACS packages by comparing single-point energies, i.e., the energies evaluated for a single, fixed set of atomic coordinates. The tetrapeptide set was constructed to probe diverse placements of each amino-acid type in a peptide sequence (details in the Methods Section).

Figure 2 summarizes unsigned single-point energy differences between GROMACS and Amber simulation engines averaged for each amino-acid type. The energies are decomposed by interaction type: the upper panel shows bonded interactions, the lower panel nonbonded. All the values were normalized to represent energies per interaction.

**Figure 2:**
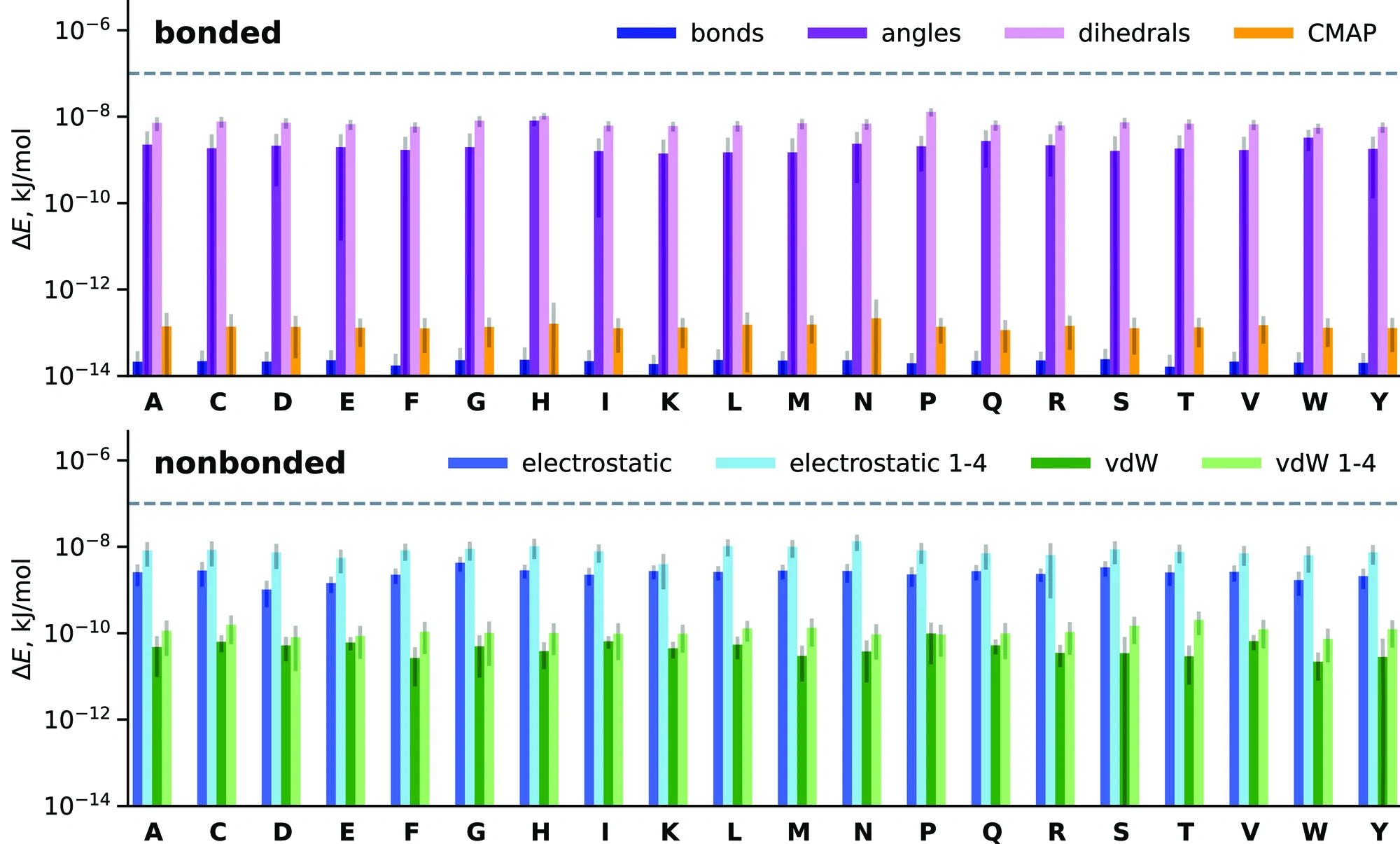
Single-point energy differences Δ*E* between Amber and GROMACS packages for each of the canonical amino acids, evaluated with the ff19SB FF. Amino acids were placed at different positions in tetrapeptides, and Δ*E* values were averaged over multiple constructs: each amino acid appeared in 78 sequences, while alanine appeared in 799 constructs. Values were normalized per interaction. Error bars indicate the standard deviation. The dashed horizontal line marks the accuracy achievable with single-precision floating-point operations.

Overall, for any interaction type, the difference in energies between the MD packages falls below the 10^–7^ kJ mol^–1^ mark, which is the accuracy level achievable for simulations performed with single-precision floating-point operations. This means that for standard MD simulations performed in single precision, we would not detect any difference in single-point energies between the simulation engines.

Across amino-acid types, the energy differences show only small variations. In contrast, across interaction types, the energy differences vary substantially, with some interaction types showing larger discrepancies. The energy difference between Amber and the GROMACS ff19SB FF port is smallest for the bond and CMAP terms, while the angle and dihedral terms show larger discrepancies. Among the nonbonded interactions, van der Waals interactions show smaller energy differences between packages compared to electrostatics.

Figure 2 showcases the final optimized version of the ff19SB FF port to GROMACS. To reach this level of numerical agreement, we needed to resolve a number of inconsistencies between the MD packages, as well as discrepancies in the interpretation of the FF parameters by the MD engines. In the next subsections we shall outline the main developments that were required for accurate energy matching between Amber and GROMACS.

#### 3.4.1 Angles: Degree-to-Radian Conversion

After the automated FF conversion to GROMACS format, significant differences in angle and dihedral energies between the Amber and GROMACS packages remained. We found that Amber’s topology generator, LEaP, used a hard-coded degree-to-radian factor (0.0174533) instead of π/180 as in GROMACS. As shown in Figure 3, replacing this constant with a more precise value significantly reduced the energy mismatch.

**Figure 3:**
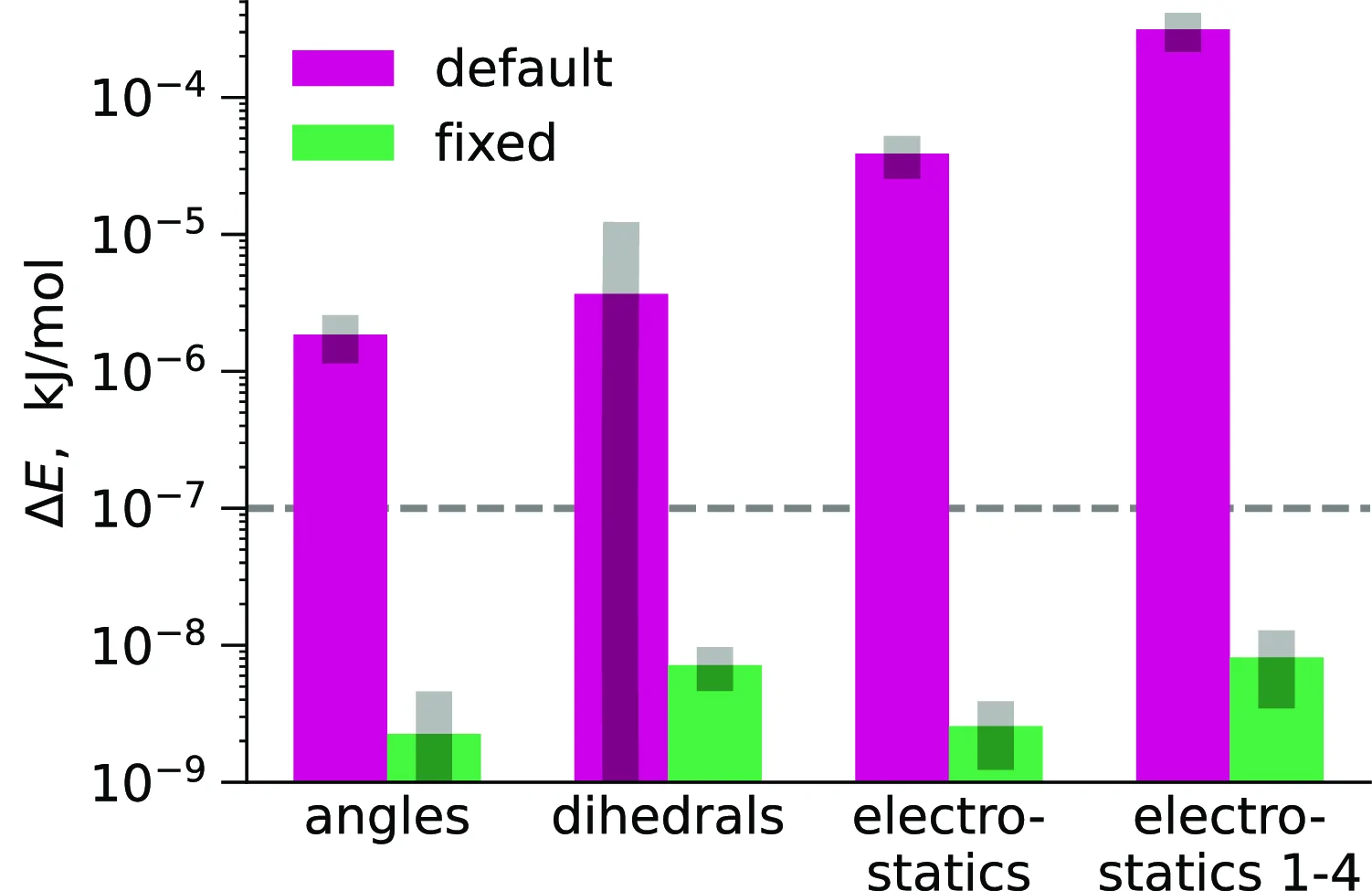
Improvements in energy matching upon fixing specific discrepancies between MD engines. Average energy difference Δ*E* per interaction between Amber and GROMACS using ff19SB, over all 20 amino acids and all 799 tetrapeptides in the set. The bars depict the following corrections: angles: degree-to-radian; dihedrals: degree-to-radian and atom ordering in improper dihedrals; electrostatics: electric conversion factor; electrostatics 1–4: ‘fudge’ factor. The dashed horizontal line marks the accuracy achievable with single-precision floating-point operations. Gray bars indicate the standard deviation.

We submitted a patch to the Amber LEaP source code to take into account the more precise conversion factor. This is consistent with newer AmberTools, such as cpptraj and ParmEd, which already use the more precise value.

#### 3.4.2 Dihedrals: Order of Impropers

The more accurate degree-to-radian conversion, however, was not sufficient to reduce the differences in dihedral energies below the single-precision limit. This discrepancy was caused by different interpretations of improper dihedrals by the MD packages, even when their definitions in the FF parameter files are identical. The Amber package dynamically reorders atoms defining improper dihedrals, and the rules of reordering depend on whether the improper dihedral has already been encountered previously. This means that the same dihedral angle may be defined differently depending on the peptide sequence (cf. Section 2.2).

We have introduced a corresponding option in GROMACS 2026 allowing improper dihedral atoms to be reordered in the same way as in the Amber package. The implementation of this new feature makes treatment of the latest Amber family FFs consistent with their original implementation in Amber.

#### 3.4.3 Electrostatics: Electric Conversion Factor

Amber LEaP has hard-coded the square root of the electric conversion factor with a value of 18.2223 (kcal mol^–1^ Å e^–2^)^1/2^. Every charge is premultiplied by this factor prior to calculating electrostatic interactions. GROMACS uses the value of 138.935458 kJ mol^–1^ nm e^–2^ for the electric conversion factor. Taking the square root and converting to the units used by Amber, results in a value of 18.2226154352 (kcal mol^–1^ Å e^–2^)^1/2^. This discrepancy is the cause for the difference in electrostatic energies as shown in Figure 3. To resolve this, we modified GROMACS to scale the topology charges by a factor that makes the resulting electrostatic energies and forces match those calculated by the Amber package. This change is controlled by a new entry in the [defaults] section of the FF. This change, like the other developments described here, has been proposed for inclusion in GROMACS. At the time of writing, however, it was not yet part of the 2026 release; we expect it to become available in a future patch or major release (Section 2.4). The energy comparisons reported in this work were obtained with a GROMACS build that includes this correction. We deliberately reproduced Amber’s value within GROMACS rather than adopting the more precise constant in Amber. Amber and the force fields parameterized for it rely on this hard-coded value, so matching it is required to reproduce Amber energies, forces, and trajectories accurately.

#### 3.4.4 Scale (‘Fudge’) Factor for Electrostatic 1–4 Interactions

Amber family FFs scale electrostatic and van der Waals interactions between the atoms separated by three bonds, i.e., 1–4 interactions. The scaling ‘fudge’ factor is expressed as 1/*f*, where *f*_Q_ = 1.2 and *f*_LJ_ = 2 in the Amber package for Coulomb and Lennard-Jones potentials, respectively. In GROMACS, however, the explicit expressions 1/*f*_Q_ = 0.8333 and 1/*f*_LJ_ = 0.5 are stored in the forcefield.itp file. The discrepancy in precision between the calculated value 1/*f*_*Q*_ = 1/1.2 and the explicitly expressed value 1/*f*_Q_ = 0.8333 results in significant energy differences (Figure 3). We expressed 1/*f*_Q_ with 17 significant digits for the two new Amber FF ports in GROMACS 2026.2 (Section 2.4). This modification brought the electrostatic 1–4 energy differences below the single-precision floating-point limit.

### 3.5 Peptide Simulation: Energy Analysis

Building on the per-amino-acid energy decomposition of the tetrapeptide set, we evaluated the converted ff19SB FF on a 27-residue peptide containing all amino-acid types, including alternative protonation states supported by ff19SB in nonterminal positions (Figure 4B). For a comprehensive assessment across a broad range of conformations, we performed MD simulations at two temperatures: 298 and 498 K. The high-temperature ensemble samples a substantially wider conformational landscape than the room-temperature ensemble (Figure 4C), covering both compact and extended geometries as well as a wider range of bond, angle, and dihedral distortions.

**Figure 4:**
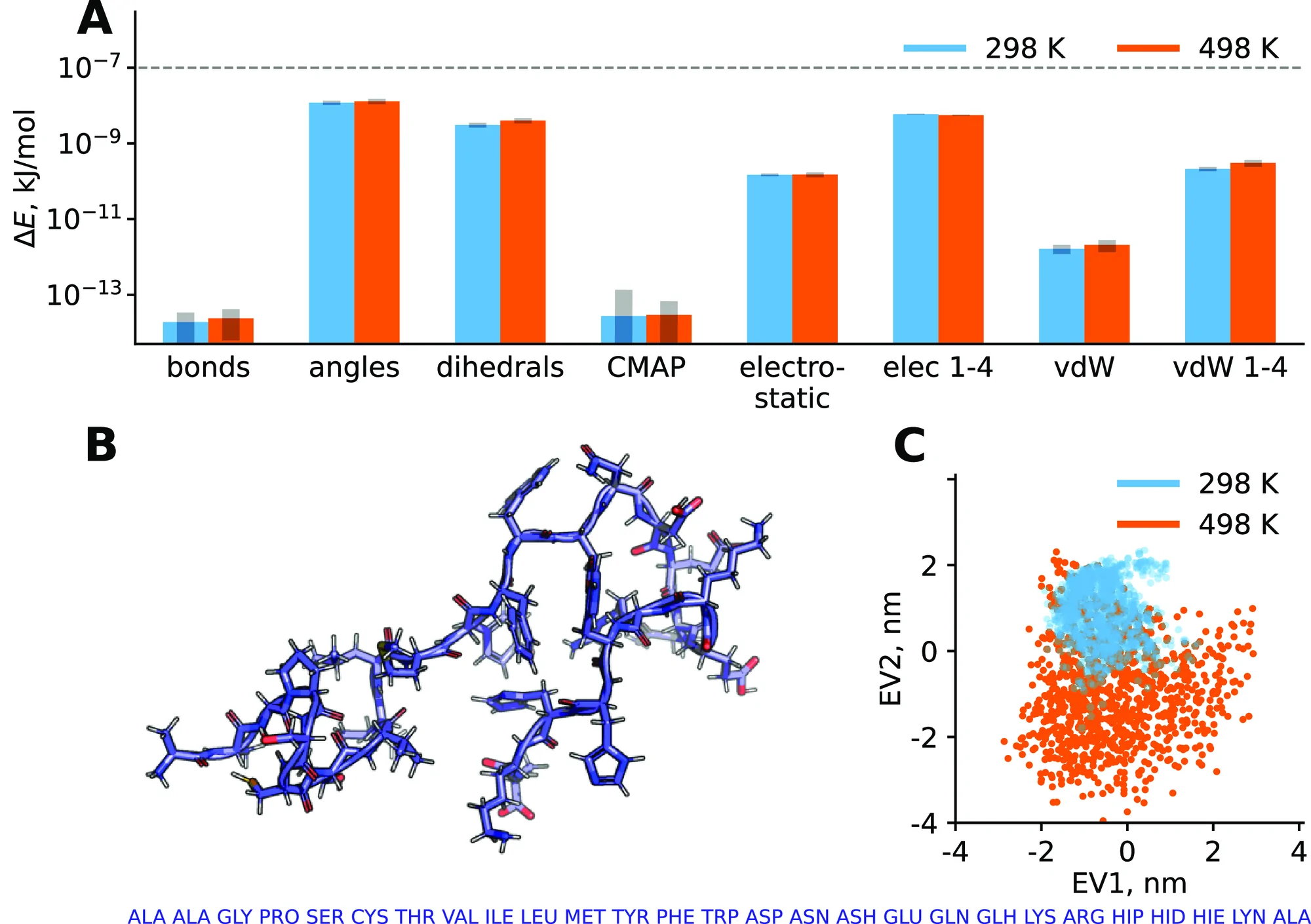
Simulations of a 27-residue peptide at room temperature and at high temperature. (A) Comparison of average energy differences Δ*E* by interaction type for conformations at 298 and 498 K; gray bars indicate the standard deviation. The dashed horizontal line marks the accuracy achievable with single-precision floating-point operations. (B) Visualization of a single peptide conformation with its amino-acid sequence shown below. (C) Comparison of the conformational landscape projected onto two principal components for MD simulations at 298 and 498 K.

Figure 4A summarizes the average per-interaction energy difference Δ*E* between Amber and GROMACS for each interaction type at the two temperatures. For all interaction types, Δ*E* remains well below the 10^–7^ kJ mol^–1^ single-precision limit, in agreement with the observations for the tetrapeptide set (Figure 2). At both 298 and 498 K, the energy differences between Amber and GROMACS are negligible, indicating that the force field port is reliable not only at the energetic minima but also across a wide range of configurations. This robustness is a prerequisite for the converted FF to behave consistently with its original Amber implementation in production MD simulations, where the peptide samples diverse conformations over time.

### 3.6 Free-Energy Profile of Ubiquitin Pincer Motion

The most fundamental requirement for a converted FF is to reproduce the single-point energies of the original FF in its native MD engine. The ultimate test, however, is whether a conformational ensemble obtained with the converted FF in GROMACS reproduces the ensemble obtained with the original FF in Amber. To address this question, we ran 10 unrestrained MD simulation replicas of ubiquitin in TIP3P water with the ff19SB FF in each engine, each 10 μs long (see Methods for the simulation protocols), as well as a control simulation with ff14SB in GROMACS.

We characterize the resulting ensembles by projecting the trajectories (backbone atoms) onto the principal component with the largest eigenvalue, which corresponds to a “pincer mode” opening and closing motion of ubiquitin (Figure 5, inset). The free-energy profile Δ*G* along this collective coordinate captures the relative populations of open and closed conformations.

**Figure 5:**
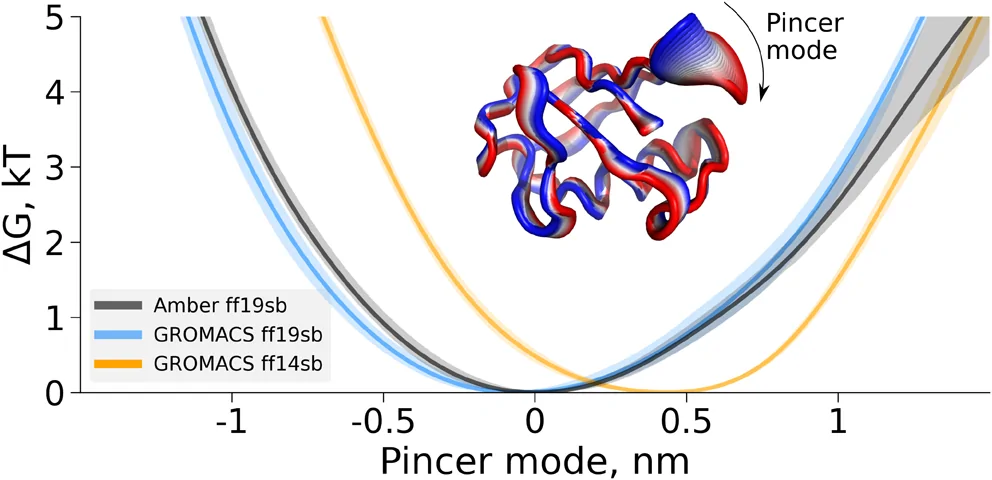
Free-energy profiles of ubiquitin along the first eigenvector from principal component analysis (the pincer mode). The Amber ff19SB (gray) and GROMACS ff19SB (blue) profiles closely overlap (shaded regions denote 95% confidence intervals), demonstrating that the converted ff19SB reproduces the conformational ensemble of the original Amber implementation. The ff14SB profile (orange) is included to illustrate the strong influence the force field has on the sampled conformational ensembles. The inset visualizes the pincer motion (red to blue color gradient).

The free-energy profiles obtained with ff19SB in Amber and GROMACS closely match across the entire range of the pincer mode (Figure 5). This indicates that the converted FF reproduces, in an ensemble-averaged sense, the behavior of the original implementation in a thermodynamic observable derived from a multimicrosecond-scale simulation. Minor deviations are to be expected due to subtle differences in the algorithms and their parameters that could not be matched exactly between the simulation engines, e.g., the neighbor-list construction algorithm and cutoffs (GROMACS dynamically adjusted neighbor list update frequency and cutoff not to exceed 0.005 kJ mol^–1^ ps^–1^ error for pair interactions per particle due to Verlet buffer), bond constraint algorithm (SHAKE for Amber and LINCS for GROMACS), differences in the pressure coupling algorithms (Monte Carlo barostat for Amber, c-rescale for GROMACS). For comparison, the older-generation force field GROMACS ff14SB produces a free-energy profile that is clearly shifted from the ff19SB profiles. This illustrates the level of variation introduced by changing the FF compared to the differences due to the MD engines when using the same FF.

## 4 Summary and Conclusions

We developed a workflow to convert recent Amber family force fields to GROMACS format. In addition, we enabled amino-acid-specific CMAP corrections in GROMACS. The ported versions of Amber ff19SB and ff14SB, together with the enhanced CMAP corrections, are part of the official GROMACS 2026 release. The correctness of the conversion was verified by comparing topologies, interaction energies, and free-energy profiles of diverse biomolecular systems simulated with the Amber and GROMACS packages.

The validation revealed several differences between the simulation engines and their interpretation of FF parameters, including different numerical precisions and values of fundamental constants, as well as the reordering of atoms in improper dihedrals. To resolve these issues, we modified the GROMACS package and Amber LEaP. The GROMACS changes have been integrated into GROMACS 2026, while the Amber patch has been submitted upstream for inclusion in a future AmberTools release.

For the tetrapeptide and 27-residue peptide validation sets, Amber and GROMACS topologies match exactly, and per-interaction energy differences remain below the single-precision limit (<10^–7^ kJ/mol). Across 10 replicas of unrestrained 10 μs ubiquitin simulations, the converted ff19SB in GROMACS reproduces the Amber free-energy profile, with closely overlapping confidence intervals. The port also includes consistent water-model and ion parameters for ff14SB and ff19SB, and the fully automated validation workflow is publicly available for future Amber FF conversions.

## Data Availability

Force field conversion and validation workflow is available at https://gitlab.mpcdf.mpg.de/barnsteen/workflow.
